# Optimization of Radium-223 Treatment of Castration-resistant Prostate Cancer Based on the Burden of Skeletal Metastasis and Clinical Parameters

**DOI:** 10.1093/oncolo/oyac245

**Published:** 2023-01-18

**Authors:** Ahmad Shariftabrizi, Shalin Kothari, Saby George, Kristopher Attwood, Ellis Levine, Dominick Lamonica

**Affiliations:** Division of Nuclear Medicine, Department of Radiology, University of Iowa Carver College of Medicine, Iowa City, IA, USA; Division of Nuclear Medicine, Department of Radiology, Roswell Park Comprehensive Cancer Center, Buffalo, NY, USA; Department of Medicine, Roswell Park Comprehensive Cancer Center, Buffalo, NY, USA; Department of Internal Medicine, Section of Hematology, Yale University School of Medicine, New Haven, CT, USA; Department of Medicine, Roswell Park Comprehensive Cancer Center, Buffalo, NY, USA; Department of Biostatistics and Informatics, Roswell Park Comprehensive Cancer Center, Buffalo, NY, USA; Department of Medicine, Roswell Park Comprehensive Cancer Center, Buffalo, NY, USA; Division of Nuclear Medicine, Department of Radiology, Roswell Park Comprehensive Cancer Center, Buffalo, NY, USA

**Keywords:** radium-223, castration-resistant prostate cancer, quantitative bone scan

## Abstract

**Background:**

Radium-223 dichloride (Ra-223) is now frequently used to treat prostate cancer that has metastasized to bone, although patient selection continues to be suboptimal for determining who will benefit most from this novel treatment modality.

**Materials and Methods:**

Seventy-nine patients with metastatic castration-resistant prostate cancer (mCRPC) were treated with Ra-223 from 2012 to 2016. The burden of skeletal metastasis was determined for each using the Bone Scan Index (BSI) as a ratio of diseased to normal bone. Clinical, laboratory, and survival data were collected and examined for associations with BSI, and treatment tolerability was assessed.

**Results:**

Chemotherapy-naïve patients were significantly more likely to complete the full course of treatment. Median follow-up was 31 months (range 0.7-38.8 months) and median overall survival was 15.4 months (range 9.5-20.6 months). Overall survival was significantly associated with findings on bone scans (*P < .*05). Patients with higher BSI tended toward poorer outcomes. Nearly half the patients with low baseline BSI survived 3 years or more following Ra-223 treatment. By contrast, only 20% of the patients with high baseline BSI lived for 1 year, and none lived for an additional 3. Baseline BSI was significantly associated with decreased hemoglobin, higher serum PSA and alkaline phosphatase levels, and treatment-associated reductions in platelet and absolute neutrophil counts.

**Conclusion:**

Our results suggest better outcomes to Ra-223 therapy for patients who are chemotherapy-naïve and who undergo treatment earlier in the course of their disease as reflected by low BSI and concordant laboratory parameters.

## Teaser

Patient selection methods are suboptimal for determining who will benefit most from Ra-223 treatment for prostate cancer that has metastasized to bone. This article characterizes bone scintigraphy as a potentially useful and important biomarker for patient selection and prognosis.

**Implications for Practice**

It is not yet entirely clear who will benefit most from Ra-223 in the context of total burden of osseous metastasis, osseous metastasis to specific regions, and underlying hematopoietic function. Based on the results of this retrospective analysis in a well-defined group of patients with castration-resistant prostate cancer, a better outcome to radium 223 therapy can be expected in chemotherapy-naïve patients with a lower BSI score, making such patients optimal candidates for this approach.

## Introduction

In 2020, the National Cancer Institute (NCI)-sponsored Surveillance, Epidemiology, and End Results (SEER) program reported that prostate cancer was the third most diagnosed cancer (191 930 cases) and led to approximately 33 330 cancer-related deaths among men in the US. Lethality is highest in cases of metastatic castration-resistant disease, suggesting an advanced clinical state and poor prognosis. As metastatic castration-resistant prostate cancer (mCRPC) is a lethal disease, the primary goal of treatment is to increase survival and enhance the quality of life.^1^

Radium-223 (Ra-223) is a first-in-class alpha-particle-emitting radiopharmaceutical currently used to treat mCRPC with overall survival benefit when used as a single agent. Ra-223 has a novel mode of action that differs from those used by chemotherapeutic agents and androgen signaling inhibitors and has a relatively benign, non-overlapping toxicity profile compared to other classes of drugs used to treat advanced-stage disease.^2^ Ra-223 is a calcium-mimetic that forms complexes with hydroxyapatite in areas of increased bone turnover, for example, in osseous metastases. Ra-223 emits alpha particle radiation which has a short path length and results in high energy transfer. These properties are responsible for its localized anti-tumor effect, which is mediated by double-strand DNA breaks.^3^ Ra-223 has a half-life of 11.4 days and emits 4 alpha particles of varying energy and detectable gamma radiation. It is excreted mainly through the intestine.^4^

Ra-223 was approved for clinical use in mCRPC based on positive results from the randomized Phase III ALSYMPCA trial. In this trial, 921 patients with mCRPC with symptomatic metastatic disease limited to the skeleton were treated with either 50 kBq/kg Ra-223 (given intravenously every 4 weeks for up to 6 cycles) or a placebo. Administration of Ra-223 significantly improved overall survival (OS) which was the primary endpoint of this study; median OS was 14 months in patients who received infusions of Ra-223 and 11.2 months among those treated with placebo. The trial also revealed a tolerable safety profile. The most common adverse effects were gastrointestinal disturbances and bone pain; grades 3 and 4 hematological toxicity was detected rarely in the Ra-223-treated patient cohort. Median time to first symptomatic skeleton-related event was significantly longer in patients treated with Ra-223 (15.6 months *versus* 9.8 months). However, no imaging was mandated in the trial nor was this considered among outcome measures.^5^

Although the ALSYMPCA trial was carried out nearly a full decade ago, there are still no reliable biomarkers that can predict which individual patients might benefit from Ra-223 therapy. Similarly, important questions remain regarding the optimal timing of Ra-223 administration during the clinical course of mCRPC. It is also not clear whether re-treatment is a valid clinical option. Currently, there are no diagnostic tests that provide reliable measurements or predict therapeutic responses to Ra-233. In this study, we aimed to characterize bone scintigraphy as a potentially useful and important biomarker for patient selection and prognosis in a single center-retrospective analysis.

## Methods

### Study Population

Patients with mCRPC consecutively treated with Ra-223 from 2012 to 2016 at Roswell Park Comprehensive Cancer Center, Buffalo, NY, USA were retrospectively evaluated. All patients had progression in the face of existing hormonal therapy and if present variable limited extent of metastatic tumor in soft tissues. All patients received standard doses based on clinical and laboratory parameters. While bone scans were used to diagnose active osteoblastic lesions, pre-treatment baseline results were not used to determine eligibility for Ra-223 therapy.

The study has been approved by the institutional review board, and the need for written informed consent was waived. The study was performed in accordance with the ethical standards of the 1964 Declaration of Helsinki and its later amendments.

### Scan Evaluation and Scoring

The burden of pre-Ra223 treatment skeletal metastasis was determined as the Bone Scan Index (BSI) as described by Imbriaco et al.^6^ Briefly, the BSI is a ratio of diseased to normal bone determined using ImageTool software (University of Texas Health Science Center, San Antonio). The approximate net weight of all metastatic lesions throughout the skeleton was calculated using International Commission on Radiological Protection (ICRP) publication No 23 (Report of the Task Group on Reference Man) and expressed as the total body BSI Time-Zero (BSI0). Clinical, laboratory, and survival data were obtained from the medical records to facilitate the evaluation of the potential links between pre-treatment BSI0 at the start of therapy, treatment tolerability, and outcomes. The skeleton was divided into 4 regions with the following designations: BSI Time-Zero Pelvis (BSI0P), BSI Time-Zero Vertebrae (BSI0V), BSI Time-Zero Ribs/Clavicle/Scapulae (BSI0RCS), and BSI Time-Zero Long Bones and Skull (BSI0LBS); these regional measurements were evaluated individually to determine the efficacy of therapy.

### Statistical Analysis

**Figure 1.**
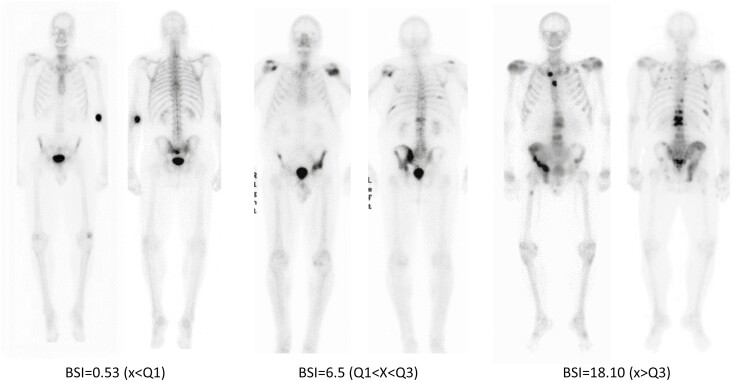
Examples of BSI values as measure of overall and regional disease burden. Cutoff values for the whole-body BSI (BSI0) quartiles: Q1-1.049/Q3-15.977.

Patient demographics, clinical characteristics, and BSI measurements were reported using mean, median, and standard deviation for continuous variables and frequencies and relative frequencies for categorical variables. Laboratory parameters were reported by Ra-233 treatment number. The association between laboratory parameters and baseline BSI values was assessed using linear mixed models where each laboratory parameter was modeled as a function of selected BSI measurement, time, their interactions, and random subject effect. Tests focused on the appropriate contrasts from the model estimates were used to evaluate (a) the main effect for each BSI measurement and (b) interactions with time (ie, whether the changes in each laboratory parameter over time were associated with baseline BSI values). All models were evaluated graphically using residual and quantile–quantile plots; transformations were applied as appropriate. Relationships were also presented graphically using line plots based on fitted regression models, in which curves for the estimated mean laboratory values over time were generated for low, average, and high baseline BSI values (Figure 1):

- Low baseline BSI (<Q1): based on the average BSI value for samples falling below the first quartile.
- Average baseline BSI (Q1-Q3): based on the average BSI value for the samples between the first and third quartile.
- High baseline BSI (>Q3): based on the average BSI value for samples falling above the third quartile.

Table 1 shows the BSI cutoff values for each quartile and the related body region. OS was defined as the time from first Ra-233 treatment until death due to any cause or last follow-up. OS was reported by baseline BSI value (<Q1, Q1-Q3, and >Q3) using standard Kaplan-Meier methods, in which estimates of the median OS and 1/3-year OS rates were obtained with 95% CIs. Comparisons were made using the log-rank test. The number of Ra-233 treatments was summarized by prior chemotherapy status and compared using the Wilcoxon exact test. All analyses were performed using Statistical Analysis Software (SAS) v9.4 (Cary, NC) at a significance level of 0.05; thus, *P*-values less than .05 denoted statistically significant results.

**Table 1.** A statistical description of the regional distribution of metastases; and Q1 and Q3 cutoff values for regional and total BSI measurements

Variable	Q1 cutoff value	Q3 cutoff value	Mean/Std/N	Median/Min/Max
BSI0P	0.059	4.062	2.7/3.3/79	1.2/0.0/8.5
BSI0V	0.375	8.565	3.6/4.2/79	1.2/0.0/11.0
BSI0RCS	0.096	4.125	2.9/4.2/79	0.5/0.0/10.6
BSI0LBS	0	5.419	7.8/14.3/79	0.6/0.0/41.5
BSI0	1.049	15.977	16.9/24.7/79	5.0/0.0/70.8

BSI0P: Pelvis Bone Scan Index at time zero, BSI0V: Vertebrae Bone Scan Index at time zero, BSI0RCS: Ribs/Clavicle/Scapulae Bone Scan Index at time zero, BSI0LBS: Long Bones and Skull Bone Scan Index at time zero.

Abbreviations: Std, Standard deviation; N, number; Min, Minimum; Max, Maximum.

## Results

Seventy-nine patients with mCRPC were treated with Ra-223 from 2012 to 2016 at Roswell Park Comprehensive Cancer Center, Buffalo, NY, USA were retrospectively evaluated. The median patient age at initiation of Ra-223 therapy was 71 years (range 46-91 years). Eighty-five percent of patients were at a Gleason score of 7 or higher and 51 patients (65%) presented with serum levels of prostate-specific antigen (PSA) >20. Nearly half of patients had undergone previous chemotherapy. Forty-nine patients (62%) completed the full 6 injections of Ra-223. Patient demographic and clinical characteristics are presented in Table 2 and BSI measurements are summarized in Table 1. A summary of the laboratory parameters by treatment number is as shown in Supplementary Table S1. The baseline BSI values were all significantly associated with measures of Hgb, platelet counts, PSA, and ALP. Higher BSI values were associated with increased levels of Hgb, PSA, and ALP. There was a significant interaction between BSI and platelet counts. Higher BSI values were initially associated with increased platelet counts, but in patients who were treated with Ra-233 (over time), higher BSI values were then associated with lower platelet counts. There was also a significant interaction term for absolute neutrophil count (ANC). We found that the ANC for the high baseline BSI group tended to decrease with time; this was not the case among those in the low or average BSI groups. The results for the regression models (main effect and interactions) indicating the above data are presented in Supplementary Table S2, which refers to the results of the linear mixed model in which the familywise error rate within a given model was controlled. These interactions are also illustrated in Supplementary Figs. S1-S5, where the fitted regression lines for low, average, and high baseline BSI values are presented for each laboratory parameter.

**Table 2.** Patients’ demographic and clinical characteristics (*N* = 79).

Characteristic	*n* (%)^a^
Age at diagnosis (years)	
Mean/Std/N	65.1/9.1/79
Median/Min/Max	66.0/43.0/90.0
Age at initiation of Ra-223 therapy (years)	
Mean/Std/N	72.1/8.7/79
Median/Min/Max	71.0/46.0/91.0
Race	
White	71 (89.9%)
Black	6 (7.6%)
Other	2 (2.5%)
Gleason score	
<7	12 (15.4%)
=7	25 (32.1%)
>7	41 (52.6%)
PSA (ng/mL) at initiation of Ra-223	
Mean/Std/N	144.0/392.7/79
Median/Min/Max	27.9/0.02/3216.1
PSA (ng/mL) at initiation	
<10	21 (26.6%)
10-20	13 (16.5%)
≥20	45 (57.0%)
High PSA (ng/mL)	
Mean/Std/N	312.0/1053.1/78
Median/Min/Max	41.1/0.1/7906.0
High PSA (ng/mL)	
<10	14 (17.9%)
10-20	13 (16.7%)
≥20	51 (65.4%)
Prior chemotherapy	
No	39 (49.4%)
Yes	40 (50.6%)
Number of Ra-233 treatments received	
1	3 (3.8%)
2	11 (13.9%)
3	5 (6.3%)
4	5 (6.3%)
5	6 (7.6%)
6	49 (62.0%)

High PSA refers to the highest PSA on record for that patient at any time in their clinical history before initiating Ra-223 treatment.

^a^Data expressed as *n* (%) unless otherwise indicated.

Abbreviations: Std, standard deviation; N, number; Min, Minimum; Max, Maximum.

**Figure 2.**
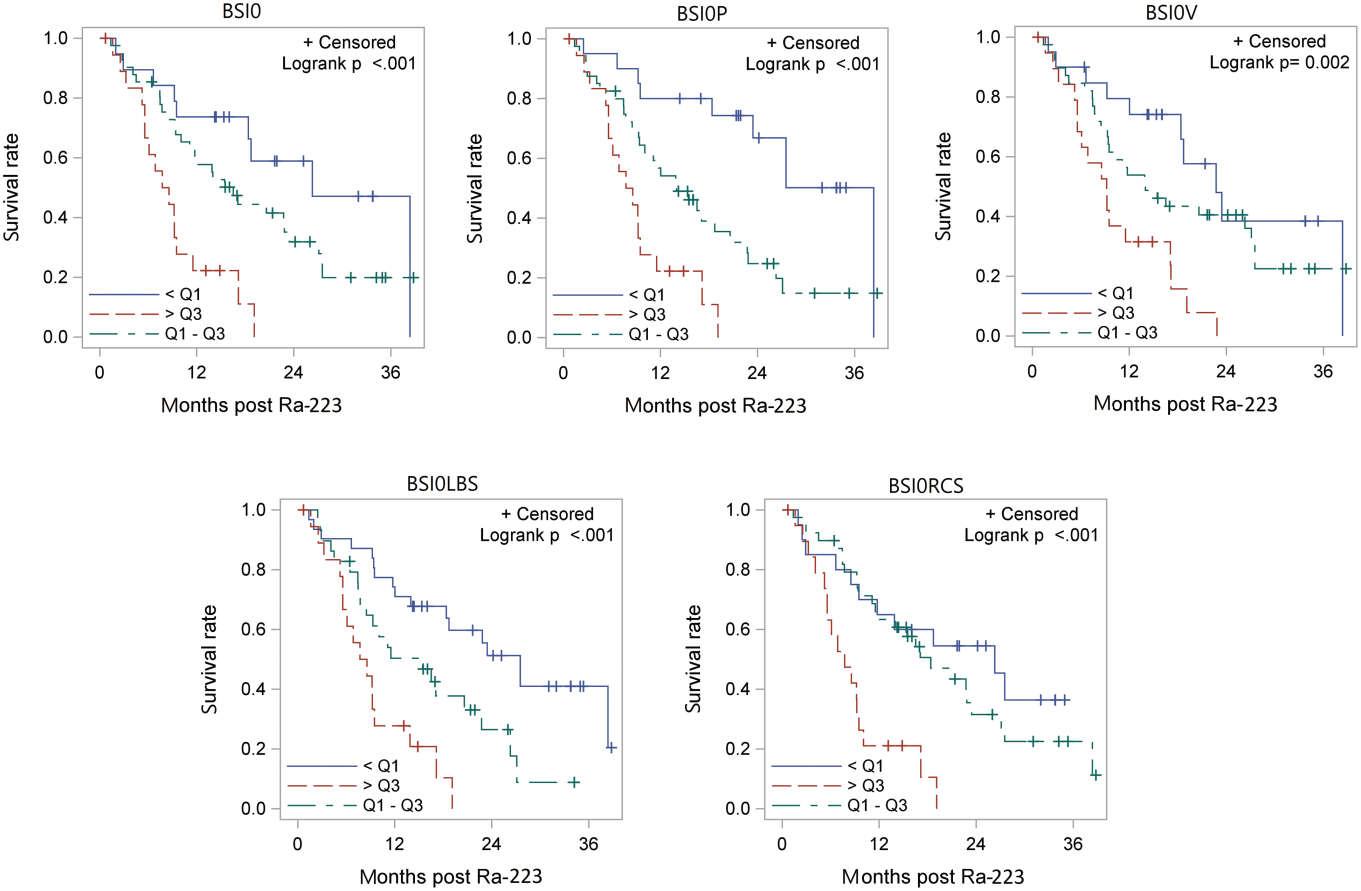
Kaplan-Meier survival based on subregional analysis of bone scans. BSI0P: Pelvis Bone Scan Index at time zero, BSI0V: Vertebrae Bone Scan Index at time zero, BSI0RCS: Ribs/Clavicle/Scapulae Bone Scan Index at time zero, BSI0LBS: Long Bones and Skull Bone Scan Index at time zero.

The OS summaries are presented in Fig. 2. OS was significantly associated with all the baseline BSIs (*P < .*05). In general, patients with higher baseline BSIs tended to have poorer outcomes (Supplementary Table S3). Results pertaining to prior chemotherapy status are presented in Supplementary Table S4. There is a significant association (*P = .*036) between patients with no prior chemotherapy and a higher number of Ra-233 treatments. Conversely, no significant association was found between baseline BSI and the number of Ra-223 cycles received (Supplementary Table S5).

## Discussion

Our combined results further support a role for determining the extent of skeletal disease in predicting treatment efficacy and tolerability of Ra-223 therapy and provide valuable insight into which patients are more likely to derive significant clinical benefit. Furthermore, in this study, we provided novel insight into the role of overall tumor burden as measured by total body BSI (BSI0) as well as the potential impact of specific regional metastases on prognosis and OS. Interestingly, we detected no differential response based on region; metastatic burden alone had a similar effect on prognosis and disease regardless of its localization both during and following Ra-223 therapy.

Few previous studies^2,7-9^ have documented a relationship between the baseline BSI and survival in subjects undergoing treatment with Ra-223. In 3 studies,^2,7,8^ the use of Ra-223 resulted in worse outcomes for individuals who presented with baseline BSI > 5. Although our results also showed that baseline BSI was significantly associated with prognosis, we did not focus on specific threshold values. Instead, by describing a stepwise association between BSI and prognosis, our analysis would indicate that the association between BSI and survival is more of a continuum with contrasting levels where patients do very poorly or very well, but also intermediate range measurements where mixed results are observed. Using this method, we identified significant differences in survival between the 3 groups. A study by Anand et al^9^ also did not propose a cutoff value for BSI, but on bivariate analysis found that ALP (and not BSI) was an indirect measure of overall tumor burden and a predictor for OS.

In our study, chemotherapy-naïve patients were more likely to survive following Ra-223 therapy and were more likely to complete all 6 treatments. This is similar to the findings of Alva et al^2^ in their study of heavily pre-treated patients, and most likely results from chemotherapy-associated marrow toxicity and lower Eastern Cooperative Oncology Group (ECOG) status in this patient subgroup. According to recommendations of the European Medicines Agency and Pharmacovigilance Risk Assessment Committee, Ra-223 treatment should be started only after the failure of 2 different systemic regimens and the presence of ≥6 osseous metastases.^10^ National Comprehensive Cancer Network Guidelines support use of Ra-223 as an alternative option for mCRPC with symptomatic bone metastases before or after docetaxel chemotherapy or novel hormonal therapy.^11^ Our results propose that Ra-223 would be most effective in chemotherapy naïve patients with lower burden of osseous metastasis.

Patients with high total BSI values were most likely to exhibit reduced platelet counts and significant reductions in other laboratory parameters, resulting in the discontinuation of therapy. Although >Q3 patients exhibited irregularities in all laboratory parameters, reductions in platelet counts and ANCs were much more pronounced and sudden in this group than in any of the other patient cohorts. This observation may relate both to the degree of treatment-associated hematopoietic dysfunction in addition to the higher osseous metastatic burden.

Patients with higher BSI values experienced increases in serum PSA during Ra-223 therapy. By contrast, those assigned to <Q1 and Q1-Q3 groups exhibited negligible PSA shifts. These findings are not consistent with those reported by Alva et al^2^ which documented no increases in PSA associated with BSI. In the ALSYMCA study, a decline in ALP levels during Ra-223 therapy was associated with improved OS.^5^ While Alva et al^2^ did not reproduce these results, we found that serum ALP levels did not decrease in the >Q3 group but remained constantly elevated throughout. This is also compatible with the results of Arnand et al^9^ showing that post-Ra223 decline in ALP is associated with longer survival. In an exploratory analysis of alkaline phosphatase dynamics in the Phase III ALSYMPCA trial, Radium-223 treated patients with ALP decline from baseline to week 12 had 55% lower risk of death compared to the patients with no confirmed decline in ALP.^12^ Similarly, van der Doelen et al^13^ have recently shown that lack of ≥10% decline in ALP following the first injection of Ra-223 may be a significant early indicator of treatment resistance.

Based on our results, a poor response to Ra-223 can be predicted during treatment in patients who experience no decline in serum ALP and/or who exhibit a rapid drop in platelet counts in conjunction with increasing PSA. These findings are typical of many patients with high baseline BSI values and would suggest that re-treatment with Ra-233 would be of little value and thus inadvisable for patients in the >Q3 cohort. This conclusion is in line with the results of a recent large clinical trial comparing standard-dose, high-dose and extended-schedule (up to 12 cycles) Ra-223 treatment, which could find no advantage in terms of overall survival or other efficacy endpoints for high-dose or extended-schedule regimens.^14^ An additional important implication of these findings is that they may provide added insight into the optimum timing of Ra-223 therapy, suggesting patients with mCRPC at earlier stages of osseous disease progression derive greatest benefit from Ra-223 administration.

The current study and conclusions drawn have certain limitations, and to change or guide current practice these would need to be validated in a larger prospective clinical trial. We are not certain if BSI cutoffs for Q1 and Q3 could be used clinically at this early stage and given the relatively small sample size in this study this would need to be validated in an expanded cohort. Moreover, in this proposed larger trial outcome measurements should also include analysis of reduction in skeletal related events and increase in quality of life. Lastly, it is presumed that the methodology used in this investigation would be further improved though enhanced consistency derived from count-based artificial intelligence-enabled automated region of interest generation for analysis of BSI.

## Supplementary Material

oyac245_suppl_Supplementary_Figures

oyac245_suppl_Supplementary_Tables

## Data Availability

The data underlying this article will be shared on reasonable request to the corresponding author.
