# Supplementary material for: Optimization of Radium-223 Treatment of Castration-resistant Prostate Cancer Based on the Burden of Skeletal Metastasis and Clinical Parameters
Source: Oncologist. 2023 Jan 18;28(3):246–51. doi: 10.1093/oncolo/oyac245 (PMC10020806; doi:10.1093/oncolo/oyac245)
Supplement: oyac245_suppl_Supplementary_Figures [file oyac245_suppl_supplementary_figures.docx]

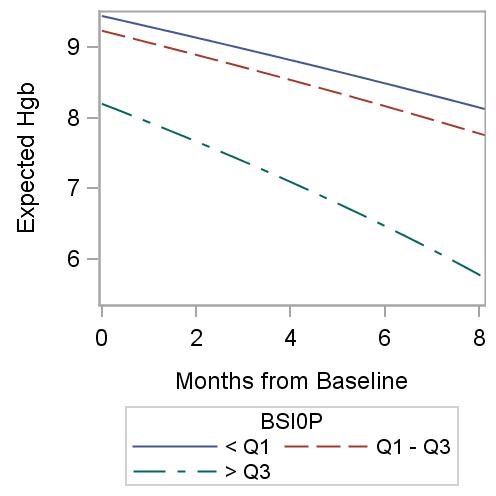


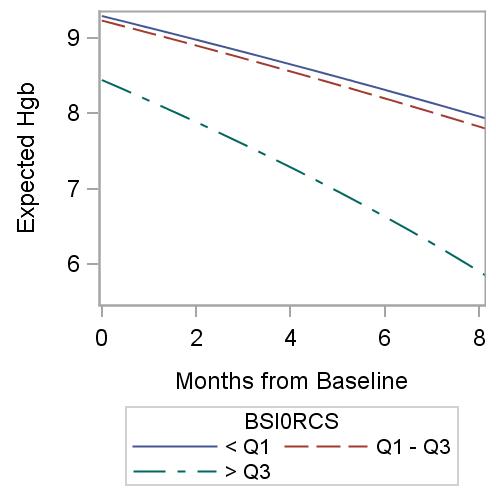

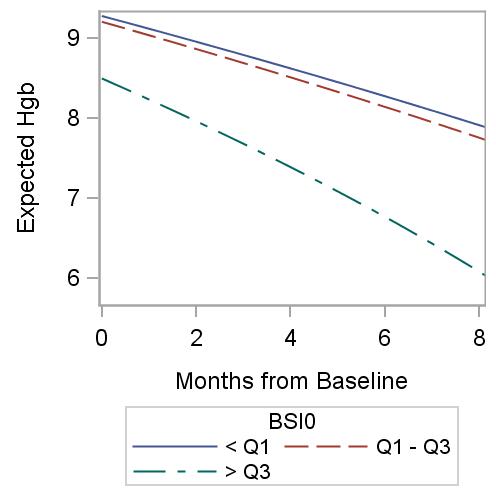


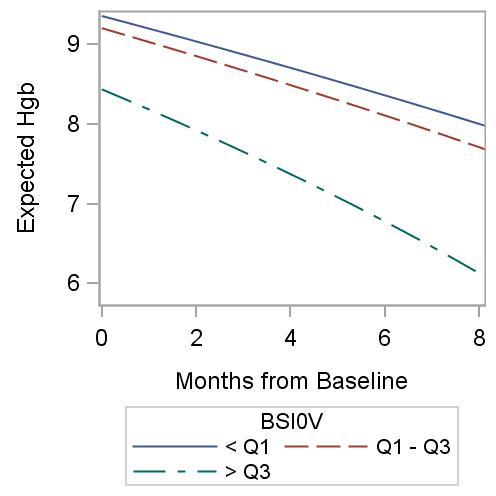

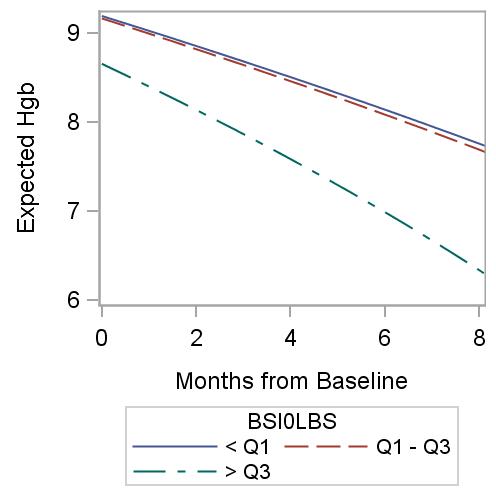


**SUPPLEMENTARY FIGURE 1.** Changes in Hemoglobin Levels Following Ra-223 therapy Based on Subregional Analysis of Bone Scans. Hgb= Hemoglobin; BSI0P= Pelvis Bone Scan Index at time zero, BSI0V= Vertebrae Bone Scan Index at time zero, BSI0RCS= Ribs/Clavicle/Scapulae Bone Scan Index at time zero, BSI0LBS= Long Bones and Skull Bone Scan Index at time zero.


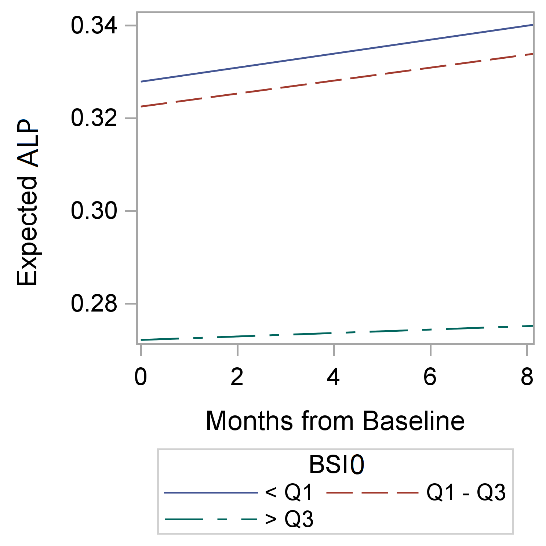

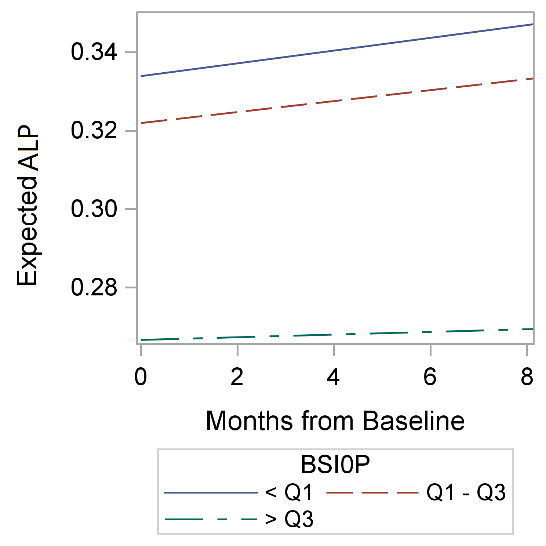

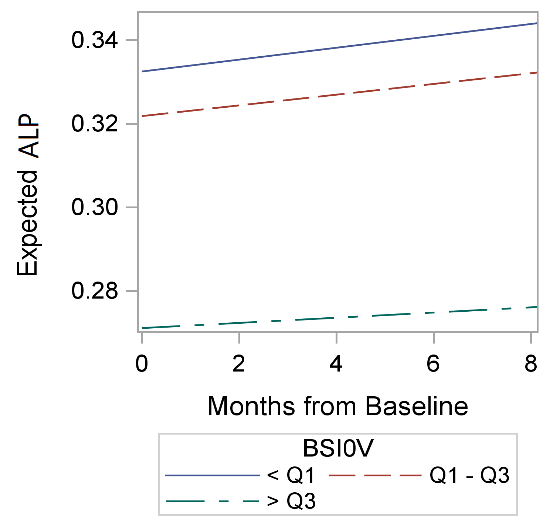


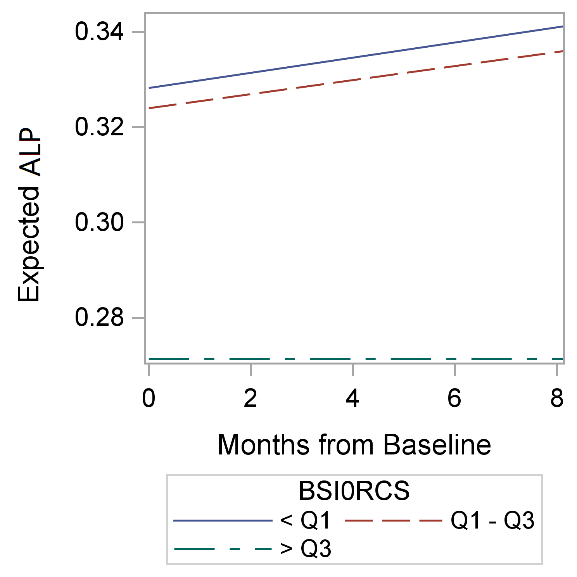


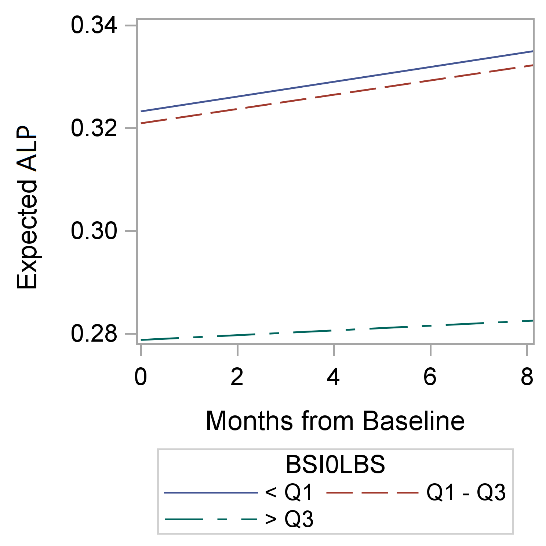


**SUPPLEMENTARY FIGURE 2.** Changes in Alkaline Phosphatase Levels Following Ra-223 Therapy Based on Subregional Analysis of Bone Scans. ALP= Alkaline Phosphatase; BSI0P= Pelvis Bone Scan Index at time zero, BSI0V= Vertebrae Bone Scan Index at time zero, BSI0RCS= Ribs/Clavicle/Scapulae Bone Scan Index at time zero, BSI0LBS= Long Bones and Skull Bone Scan Index at time zero.


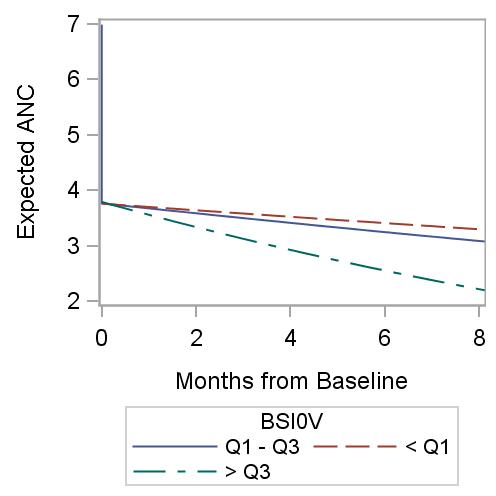

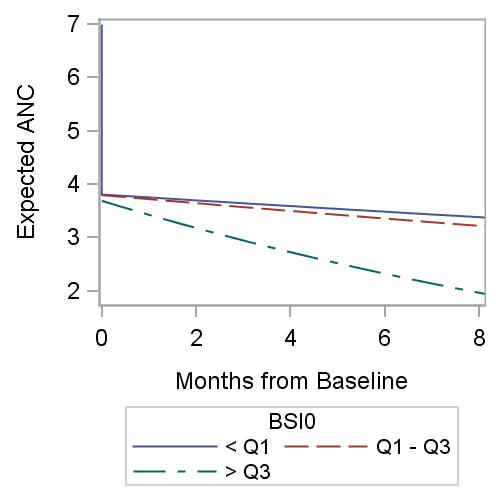

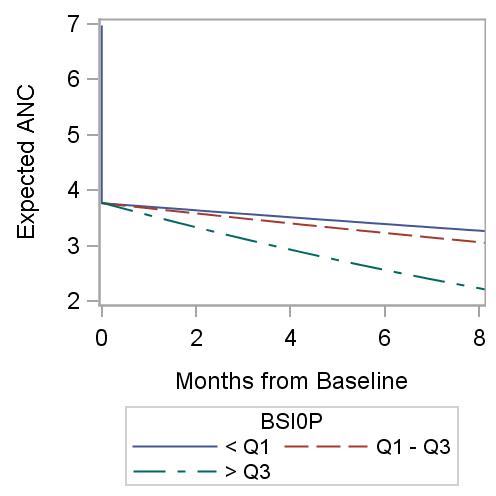


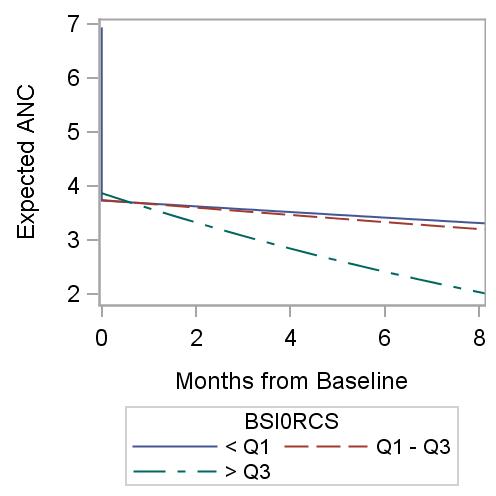

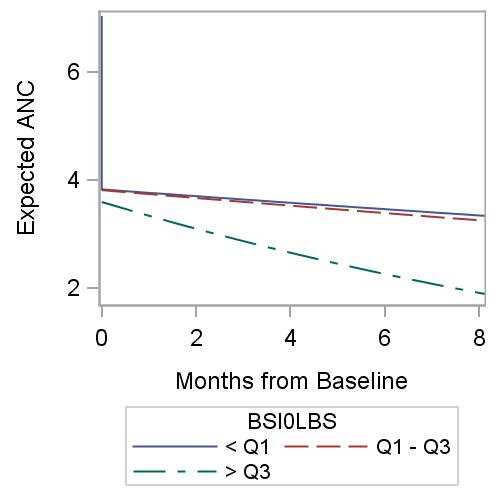


**SUPPLEMENTARY FIGURE 3.** Changes in Absolute Neutrophil Count Following Ra-223 Therapy Based on Subregional Analysis of Bone Scans. ANC= Absolute Neutrophil Count; BSI0P= Pelvis Bone Scan Index at time zero, BSI0V= Vertebrae Bone Scan Index at time zero, BSI0RCS= Ribs/Clavicle/Scapulae Bone Scan Index at time zero, BSI0LBS= Long Bones and Skull Bone Scan Index at time zero.


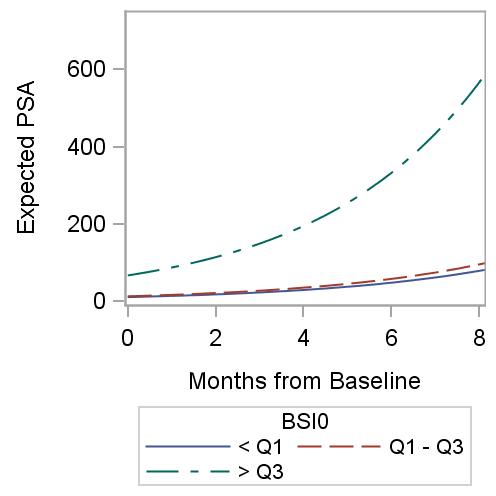

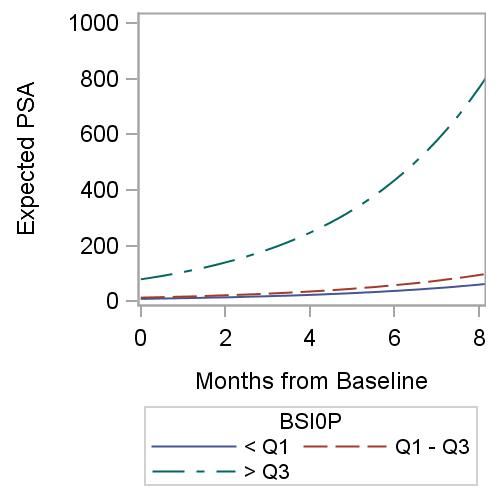

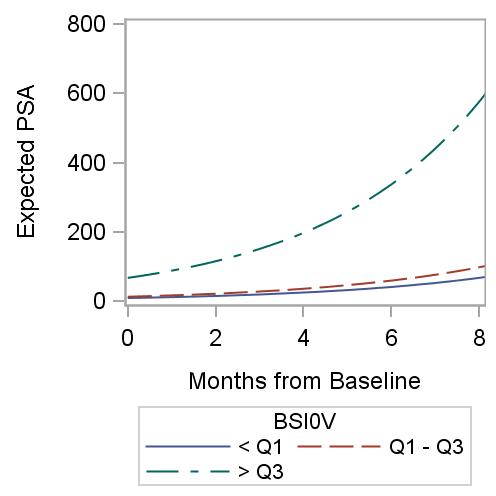


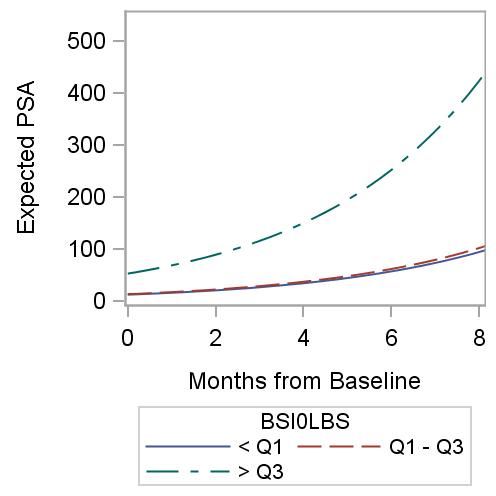


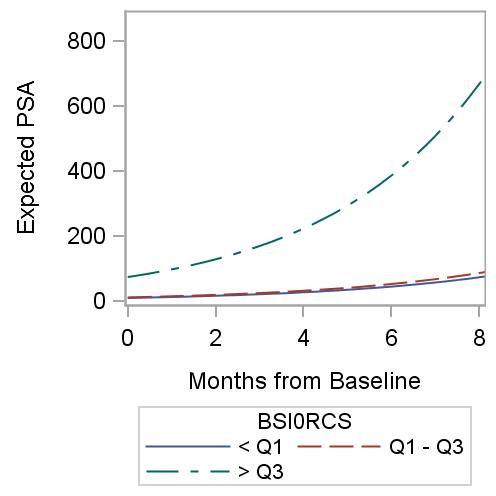


**SUPPLEMENTARY FIGURE 4.** Changes in Platelet Counts Following Ra-223 Therapy Based on Subregional Analysis of Bone Scans. PLT= Platelets; BSI0P= Pelvis Bone Scan Index at time zero, BSI0V= Vertebrae Bone Scan Index at time zero, BSI0RCS= Ribs/Clavicle/Scapulae Bone Scan Index at time zero, BSI0LBS= Long Bones and Skull Bone Scan Index at time zero.


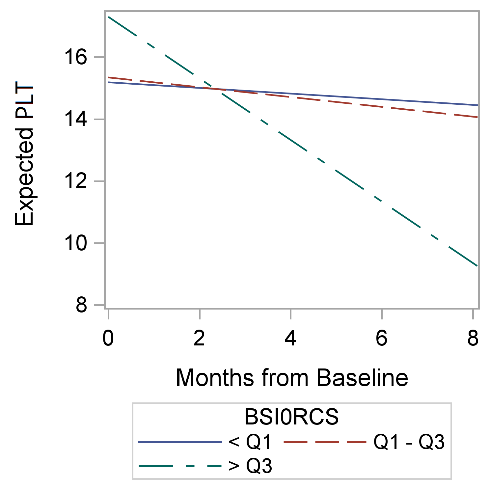

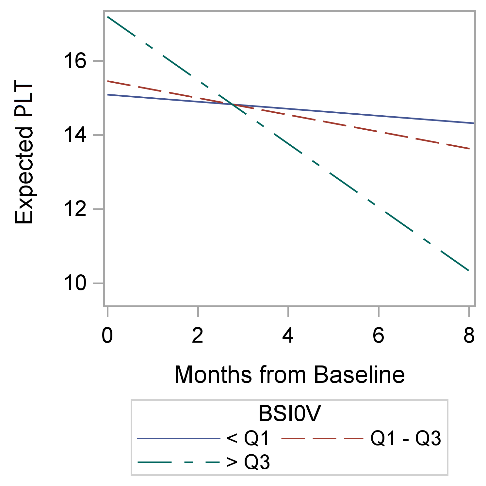

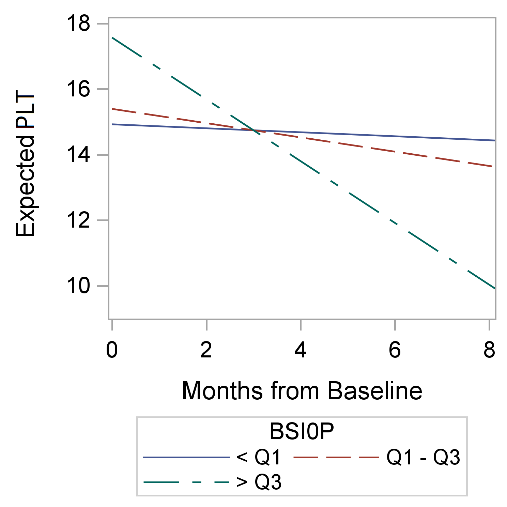

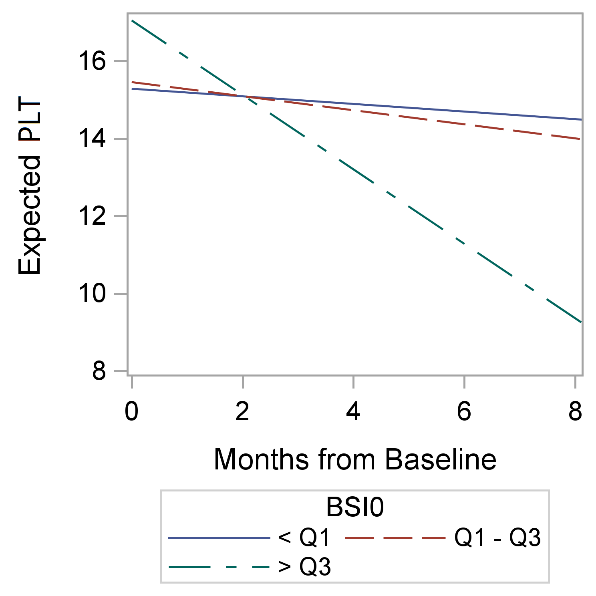


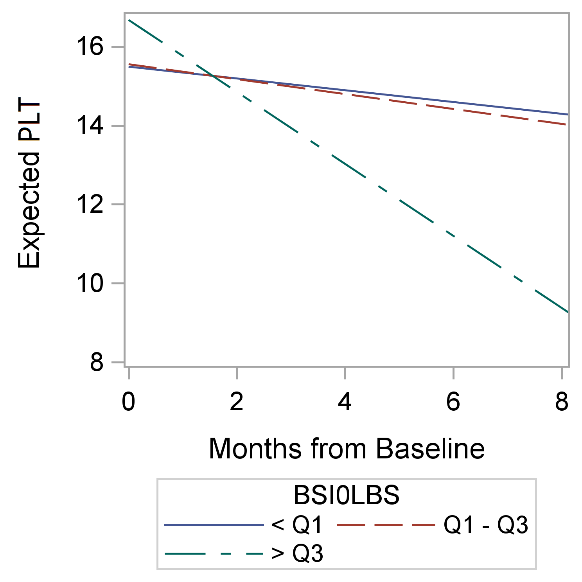


**SUPPLEMENTARY FIGURE 5.** Changes in Serum Prostate-specific Antigen Levels Following Ra-223 Therapy Based on Subregional Analysis of Bone Scans. PSA=Prostate Specific Antigen; BSI0P= Pelvis Bone Scan Index at time zero, BSI0V= Vertebrae Bone Scan Index at time zero, BSI0RCS= Ribs/Clavicle/Scapulae Bone Scan Index at time zero, BSI0LBS= Long Bones and Skull Bone Scan Index at time zero.
