# Supplementary material for: Optimization of Radium-223 Treatment of Castration-resistant Prostate Cancer Based on the Burden of Skeletal Metastasis and Clinical Parameters
Source: Oncologist. 2023 Jan 18;28(3):246–51. doi: 10.1093/oncolo/oyac245 (PMC10020806; doi:10.1093/oncolo/oyac245)
Supplement: oyac245_suppl_Supplementary_Tables [file oyac245_suppl_supplementary_tables.docx]

**Supplementary Table 1**. A Statistical Description of Laboratory Parameters at Baseline and Following each Round of Ra-223 Therapy.

|  | | **Baseline** | **Ra-233 (1)** | **Ra-233 (2)** | **Ra-233 (3)** | **Ra-233 (4)** | **Ra-233 (5)** | **Ra-233 (6)** |
| --- | --- | --- | --- | --- | --- | --- | --- | --- |
| Hgb (g/dL) | Mean/ Std/ N | 12.3/ 1.7/ 79 | 11.9/ 1.8/ 79 | 11.8/ 1.7/ 76 | 11.7/ 1.6/ 65 | 11.5/ 1.6/ 60 | 11.1/ 1.9/ 55 | 10.7/ 2.3/ 49 |
|  | Median/ Min/ Max | 12.3/ 8.0/ 15.3 | 12.1/ 6.5/ 16.0 | 11.9/ 7.4/ 14.7 | 11.9/ 8.0/ 14.6 | 11.6/ 7.7/ 14.6 | 11.2/ 6.4/ 14.2 | 11.0/ 5.3/ 14.5 |
| PLT  (k/uL) | Mean/ Std/ N | 261.0/ 88.3/ 79 | 242.3/ 86.5/ 79 | 229.7/ 87.4/ 76 | 224.0/ 71.6/ 65 | 221.1/ 93.7/ 60 | 213.4/ 92.8/ 55 | 208.8/ 100.1/ 49 |
|  | Median/ Min/ Max | 239.0/ 36.4/ 472.0 | 225.0/ 75.0/ 478.0 | 215.0/ 31.0/ 520.0 | 214.0/ 80.0/ 437.0 | 207.0/ 54.0/ 553.0 | 204.0/ 38.0/ 490.0 | 202.0/ 37.0/ 513.0 |
| ANC (k/uL) | Mean/ Std/ N | 4.6/ 1.8/ 78 | 3.5/ 1.6/ 79 | 3.5/ 1.5/ 76 | 3.5/ 1.3/ 65 | 3.5/ 1.4/ 60 | 3.5/ 1.7/ 55 | 3.7/ 1.3/ 49 |
|  | Median/ Min/ Max | 4.2/ 0.9/ 11.8 | 3.3/ 0.9/ 8.4 | 3.3/ 0.8/ 8.5 | 3.3/ 0.7/ 7.2 | 3.5/ 0.9/ 8.2 | 3.1/ 0.6/ 10.6 | 3.6/ 0.8/ 6.8 |
| PSA (ng/mL) | Mean/ Std/ N | 110.7/ 322.1/ 79 | 144.0/ 392.7/ 79 | 156.5/ 374.8/ 76 | 164.1/ 368.4/ 65 | 198.4/ 410.1/ 60 | 248.6/ 522.5/ 55 | 287.8/ 637.9/ 49 |
|  | Median/ Min/ Max | 17.7/ 0.0/ 2570.3 | 27.9/ 0.0/ 3216.1 | 39.5/ 0.0/ 2945.5 | 32.0/ 0.0/ 2496.7 | 42.7/ 0.0/ 2581.7 | 55.8/ 0.0/ 2731.3 | 53.6/ 0.0/ 3439.9 |
| ALP (u/L) | Mean/ Std/ N | 153.4/ 137.6/ 79 | 143.0/ 122.5/ 79 | 125.6/ 105.6/ 76 | 126.0/ 127.2/ 65 | 133.1/ 140.8/ 60 | 124.9/ 122.8/ 55 | 135.9/ 144.1/ 49 |
|  | Median/ Min/ Max | 110.0/ 35.0/ 924.0 | 100.0/ 5.3/ 690.0 | 91.0/ 9.3/ 527.0 | 85.0/ 37.0/ 703.0 | 92.0/ 37.0/ 830.0 | 88.0/ 37.0/ 693.0 | 80.0/ 27.0/ 771.0 |

**Abbreviations:** Hgb= Hemoglobin, PLT= Platelets, ANC= Absolute Neutrophil Count, PSA=Prostate Specific Antigen, ALP= Alkaline Phosphatase. Std=Standard deviation, N=number, Min= Minimum, Max= Maximum.

**Supplementary Table 2.** Association Between Regional Baseline Bone Scan Index Values and Laboratory Parameters as Determined by Regression Analysis.

| **Outcome: Laboratory Parameter** | **Predictor: BSI** | | **Estimated Regression Coefficients (Standard Error)** | | | **P-value** | | |
| --- | --- | --- | --- | --- | --- | --- | --- | --- |
|  |  |  | **BSI** | **Time** | **Interaction** | **BSI** | **Time** | **Interaction** |
| Hgb ^a^ | | BSI0P | -2.6581 (0.6172) | -2.8460 (0.5155) | -0.1674 (0.1228) | <.001 | <.001 | 0.174 |
|  | | BSI0V | -1.6120 (0.5081) | -2.9238 (0.5261) | -0.1243 (0.0985) | 0.002 | <.001 | 0.208 |
|  | | BSI0RCS | -1.5458 (0.5057) | -2.8707 (0.4765) | -0.1722 (0.0971) | 0.002 | <.001 | 0.077 |
|  | | BSI0LBS | -0.3191 (0.1534) | -3.0325 (0.4491) | -0.0434 (0.0282) | 0.038 | <.001 | 0.125 |
|  | | BSI0 | -0.2449 (0.0869) | -2.9195 (0.4774) | -0.0259 (0.0163) | 0.005 | <.001 | 0.114 |
| PLT ^b^ | | BSI0P | 0.3207 (0.0883) | -0.0595 (0.1218) | -0.1069 (0.0290) | <.001 | 0.627 | <.001 |
|  | | BSI0V | 0.2073 (0.0715) | -0.0864 (0.1248) | -0.0750 (0.0233) | 0.004 | 0.491 | 0.001 |
|  | | BSI0RCS | 0.2170 (0.0703) | -0.0886 (0.1076) | -0.0926 (0.0219) | 0.002 | 0.413 | <.001 |
|  | | BSI0LBS | 0.0394 (0.0215) | -0.1492 (0.1026) | -0.0254 (0.0064) | 0.067 | 0.150 | <.001 |
|  | | BSI0 | 0.0311 (0.0122) | -0.0910 (0.1080) | -0.0152 (0.0037) | 0.011 | 0.402 | <.001 |
| ANC ^c^ | | BSI0P | 0.0001 (0.0049) | -0.0060 (0.0038) | -0.0019 (0.0009) | 0.984 | 0.121 | 0.042 |
|  | | BSI0V | 0.0003 (0.0038) | -0.0054 (0.0038) | -0.0016 (0.0007) | 0.942 | 0.162 | 0.028 |
|  | | BSI0RCS | 0.0013 (0.0038) | -0.0050 (0.0034) | -0.0021 (0.0007) | 0.735 | 0.145 | 0.002 |
|  | | BSI0LBS | -0.0007 (0.0011) | -0.0058 (0.0031) | -0.0006 (0.0002) | 0.518 | 0.068 | 0.001 |
|  | | BSI0 | -0.0002 (0.0006) | -0.0049 (0.0034) | -0.0004 (0.0001) | 0.762 | 0.148 | 0.003 |
| PSA ^d^ | | BSI0P | 0.2620 (0.0673) | 0.2360 (0.0414) | 0.0058 (0.0098) | <.001 | <.001 | 0.558 |
|  | | BSI0V | 0.1922 (0.0537) | 0.2439 (0.0420) | 0.0023 (0.0078) | <.001 | <.001 | 0.771 |
|  | | BSI0RCS | 0.2009 (0.0527) | 0.2431 (0.0385) | 0.0031 (0.0078) | <.001 | <.001 | 0.690 |
|  | | BSI0LBS | 0.0474 (0.0161) | 0.2494 (0.0365) | 0.0003 (0.0023) | 0.003 | <.001 | 0.892 |
|  | | BSI0 | 0.0318 (0.0091) | 0.2458 (0.0387) | 0.0004 (0.0013) | <.001 | <.001 | 0.785 |
| ALP ^e^ | | BSI0P | -0.0082 (0.0015) | 0.0016 (0.0013) | -0.0002 (0.0003) | <.001 | 0.227 | 0.626 |
|  | | BSI0V | -0.0060 (0.0012) | 0.0014 (0.0014) | -0.0001 (0.0003) | <.001 | 0.297 | 0.758 |
|  | | BSI0RCS | -0.0058 (0.0012) | 0.0016 (0.0012) | -0.0002 (0.0003) | <.001 | 0.206 | 0.528 |
|  | | BSI0LBS | -0.0015 (0.0004) | 0.0014 (0.0012) | -0.0000 (0.0001) | <.001 | 0.228 | 0.662 |
|  | | BSI0 | -0.0010 (0.0002) | 0.0015 (0.0013) | -0.0000 (0.0000) | <.001 | 0.231 | 0.643 |

The column labeled “BSI Effect” includes the *p*-values associated with tests of the overall effect of the baseline BSIs on the different laboratory parameters. The column labeled “BS-Time Interaction” includes the *p*-values associated with the bone scan-time interaction term, which assesses whether the relationships between the laboratory parameters and time depend on the baseline bone scan.

Abbreviations: BSI= Bone Scan Index, BSI0P= Pelvis Bone Scan Index at time zero, BSI0V= Vertebrae Bone Scan Index at time zero, BSI0RCS= Ribs/Clavicle/Scapulae Bone Scan Index at time zero, BSI0LBS= Long Bones and Skull Bone Scan Index at time zero. Hgb= Hemoglobin, PLT= Platelets, ANC= Absolute Neutrophil Count, PSA=Prostate Specific Antigen, ALP= Alkaline Phosphatase.

**Supplementary Table 3.** One- and 3-year Overall Survival Based on Subregional Analysis of Bone Scans.

|  | | **1-yr OS Rate (95% CI)** | **3-yr OS Rate (95% CI)** | **Median OS (95% CI)** | **Median Follow-up Range (95% CI)** |
| --- | --- | --- | --- | --- | --- |
| Overall | | 0.55 (0.43–0.65) | 0.22 (0.12–0.34) | 15.4 (9.5–20.6) | 31.0 (0.7–38.8) |
| BSI0P | < Q1 | 0.80 (0.55–0.92) | 0.50 (0.23–0.72) | 38.4 (18.4–38.4) |  |
|  | Q1–Q3 | 0.57 (0.40–0.70) | 0.15 (0.04–0.31) | 14.0 (9.4–20.6) |  |
|  | > Q3 | 0.22 (0.07–0.43) | 0.00 (0.01–0.35) | 8.1 (5.6–9.5) |  |
| BSI0V | < Q1 | 0.79 (0.54–0.92) | 0.38 (0.13–0.64) | 22.7 (12.0–38.4) |  |
|  | Q1–Q3 | 0.54 (0.37–0.68) | 0.23 (0.09–0.39) | 14.0 (9.3 –27.1) |  |
|  | > Q3 | 0.32 (0.13–0.52) | 0.00 (0.01–0.29) | 9.2 (5.6–17.1) |  |
| BSI0RCS | < Q1 | 0.65 (0.40–0.82) | 0.36 (0.13–0.60) | 26.3 (8.5–NR) |  |
|  | Q1–Q3 | 0.66 (0.49–0.79) | 0.23 (0.09–0.40) | 18.4 (11.5–23.4) |  |
|  | > Q3 | 0.21 (0.07–0.41) | 0.00 (0.01–0.34) | 7.7 (5.2–9.5) |  |
| BSI0LBS | < Q1 | 0.74 (0.55–0.86) | 0.41 (0.22–0.59) | 27.5 (14.0–NR) |  |
|  | Q1–Q3 | 0.50 (0.31–0.67) | 0.09 (0.01–0.31) | 15.4 (7.7–22.7) |  |
|  | > Q3 | 0.28 (0.10–0.49) | 0.00 (0.01–0.34) | 8.1 (5.6–9.5) |  |
| BSI0 | < Q1 | 0.74 (0.48–0.88) | 0.47 (0.19–0.71) | 26.3 (9.5–38.4) |  |
|  | Q1–Q3 | 0.60 (0.44–0.73) | 0.20 (0.08–0.36) | 16.5 (10.1–23.4) |  |
|  | > Q3 | 0.22 (0.07–0.43) | 0.00 (0.01–0.35) | 8.1 (5.6–9.5) |  |

Abbreviations: OS=Overall Survival, CI= Confidence Interval. BSI0P= Pelvis Bone Scan Index at time zero, BSI0V= Vertebrae Bone Scan Index at time zero, BSI0RCS= Ribs/Clavicle/Scapulae Bone Scan Index at time zero, BSI0LBS= Long Bones and Skull Bone Scan Index at time zero.

**Supplementary Table 4.** Effect of Previous Chemotherapy on Completion of Ra-223 Therapy.

| **Ra-223 Cycles** | | **No** | **Yes** | ***P*-value** |
| --- | --- | --- | --- | --- |
|  | Total | 39 (49.4) | 40 (50.6) |  |
|  | 1 |  | 3 (7.5%) | - |
|  | 2 | 3 (7.7%) | 8 (20.0%) |  |
|  | 3 | 2 (5.1%) | 3 (7.5%) |  |
|  | 4 | 3 (7.7%) | 2 (5.0%) |  |
|  | 5 | 3 (7.7%) | 3 (7.5%) |  |
|  | 6 | 28 (71.8%) | 21 (52.5%) |  |
|  | Mean/ Std/ N | 5.3/ 1.3/ 39 | 4.4/ 1.9/ 40 | 0.036 |
|  | Median/ Min/ Max | 6.0/ 2.0/ 6.0 | 6.0/ 1.0/ 6.0 |  |

Abbreviations: Std=Standard deviation, N=number, Min= Minimum, Max= Maximum.

**Supplementary Table-5.** Association Between the Total Body Bone Scan Index at Time Zero with Number of Ra-223 Cycles.

|  | **BSIO** | **< Q1** | **Q1-Q3** | **> Q3** |
| --- | --- | --- | --- | --- |
| **Number of Ra-233 treatments received N (%)** | **1** | 0 (0%) | 2 (4.9%) | 1 (5.3%) |
|  | **2** | 2 (10.5%) | 5 (12.2%) | 4 (21.1%) |
|  | **3** | 0 (0%) | 4 (9.8%) | 1 (5.3%) |
|  | **4** | 0 (0%) | 4 (9.8%) | 1 (5.3%) |
|  | **5** | 1 (5.3%) | 2 (4.9%) | 3 (15.6%) |
|  | **6** | 16 (84.2%) | 24 (58.5%) | 9 (47.4%) |

No significant correlation was found between the total body BSI at baseline (BSI0) with the number of Ra-223 cycles received (p=0.410).
